# Modeling the influence of gravity and the mechanical properties of elastin and collagen fibers on alveolar and lung pressure–volume curves

**DOI:** 10.1038/s41598-022-16650-0

**Published:** 2022-07-19

**Authors:** Linzheng Shi, Jacob Herrmann, Samer Bou Jawde, Jason H. T. Bates, Hadi T. Nia, Béla Suki

**Affiliations:** 1 Department of Biomedical Engineering, Boston University, 44 Cummington Mall, Boston, MA 02215 USA; 2 Department of Medicine, University of Vermont, Burlington, VT USA

**Keywords:** Biomedical engineering, Computational models, Respiration

## Abstract

The relationship between pressure (P) and volume (V) in the human lung has been extensively studied. However, the combined effects of gravity and the mechanical properties of elastin and collagen on alveolar and lung P–V curves during breathing are not well understood. Here, we extended a previously established thick-walled spherical model of a single alveolus with wavy collagen fibers during positive pressure inflation. First, we updated the model for negative pressure-driven inflation that allowed incorporation of a gravity-induced pleural pressure gradient to predict how the static alveolar P–V relations vary spatially throughout an upright human lung. Second, by introducing dynamic surface tension and collagen viscoelasticity, we computed the hysteresis loop of the lung P–V curve. The model was tested by comparing its predicted regional ventilation to literature data, which offered insight into the effects of microgravity on ventilation. The model has also produced novel testable predictions for future experiments about the variation of mechanical stresses in the septal walls and the contribution of collagen and elastin fibers to the P–V curve and throughout the lung. The model may help us better understand how mechanical stresses arising from breathing and pleural pressure variations affect regional cellular mechanotransduction in the lung.

## Introduction

The mechanical properties of the human lung are embodied in the relationship between lung pressure (P) and volume (V) as represented by the P–V curve. This relationship has been extensively explored under normal and diseased conditions^1–13^ and provides an overall picture of lung health. The P–V behavior is not uniform throughout the lung, however; regional variations in specific ventilation reflect spatial heterogeneities in the mechanical characteristics of lung tissue^14^. These heterogeneities are the result of factors that manifest over a wide range of length scales. At the scale of the whole lung, changes in intrapleural pressure drive overall lung inflation during spontaneous breathing, but the intrapleural pressure itself exhibits a gravitational gradient due to the weight of the lung^14–17^. This gradient, in turn, influences regional lung expansion according to the orientation of the subject^18^. At the scale of individual alveoli, regional lung expansion is determined by the spatial distribution of alveolar compliance^19^, which determines how each alveolus expands due to a change in local transpulmonary pressure^2^. Alveolar compliance itself is determined at the scale of the alveolar septal wall by surface tension at the air–liquid interface and by the extracellular matrix (ECM) that consists predominantly of a network of collagen and elastin fibers^20,21^.

How these various factors collectively give rise to the regional mechanical properties of the lung parenchyma remains poorly understood yet is fundamental to the interpretation of normal P–V behavior. This, in turn, is crucial for understanding what may be behind the P–V changes that take place in lung diseases such as emphysema and pulmonary fibrosis, and also has significant implications for mechanotransduction in cells that are embedded within the parenchyma. The interactions between the multi-scale mechanical processes taking place in the lung, however, are complex and nonlinear, and thus their consequences cannot be properly evaluated without the aid of significant computational effort.

Accordingly, our goal in the present study is to develop a computational model of the lung that incorporates the key multi-scale processes alluded to above in a way that accurately explains the P–V behavior of the normal lung. The starting point for this study is an analytical model of the mechanical behavior of a single alveolus we recently developed^2^. This previous model represents each alveolus as a thick-walled spherical shell in which wavy collagen fibers are embedded, providing important insight into the factors that determine the inflation stability of the alveolus^2^, but it does not account for the way in which the P–V curve is impacted by regional heterogeneities due either to gravity or to inherent regional variability in intrinsic tissue mechanics. Here, we extend this single alveolus model by simulating its inflation and deflation with a negative pleural pressure subject to a gravitational gradient. This allows us to calculate how alveolar stiffness varies with vertical position in the lung, and how this translates into regional distributions of mechanical stresses in the alveolar septal wall and in the collagen and elastin fibers embedded within it.

## Results

**Figure 1:**
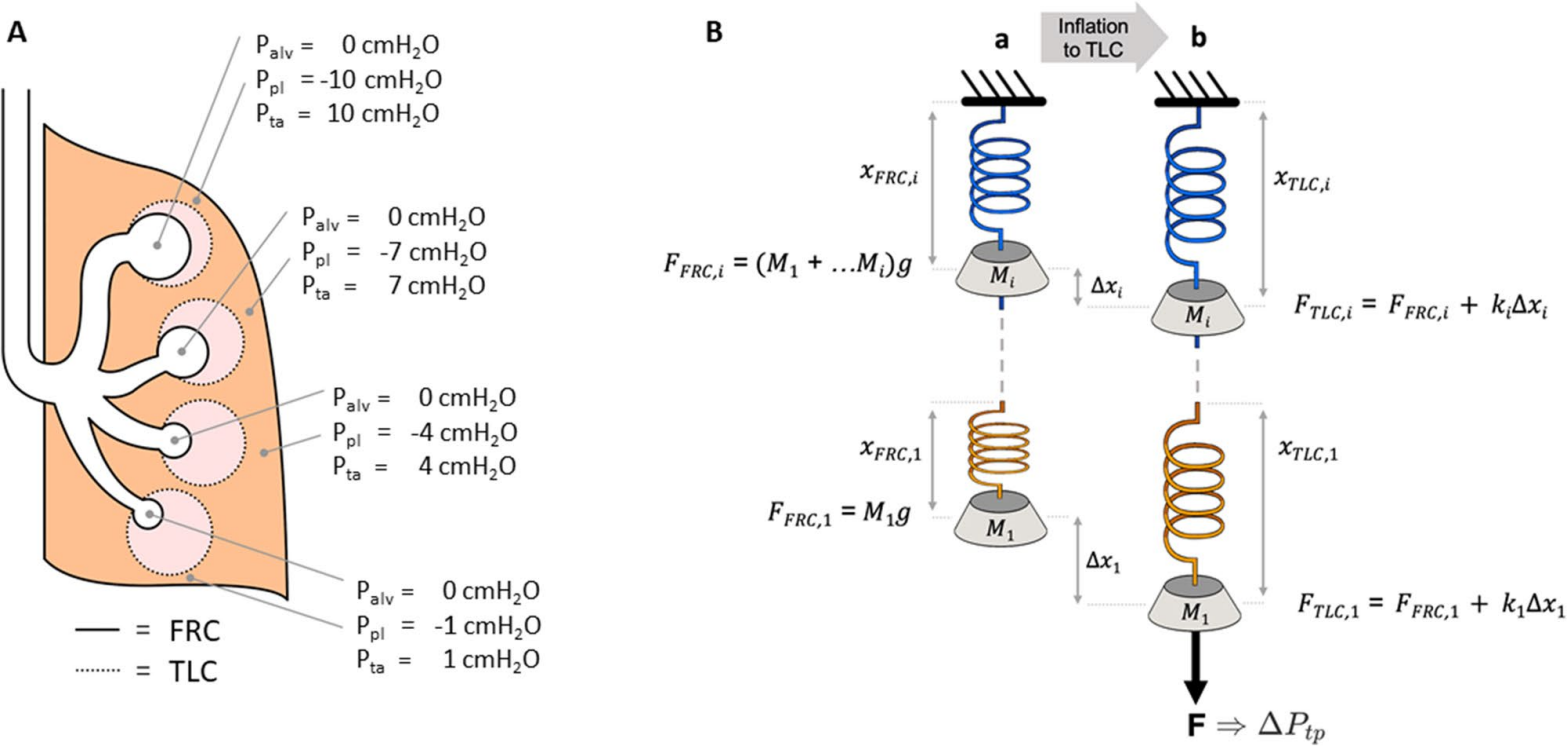
(**A**) schematic representation of the upright human lung with pleural ($P_{pl}$), alveolar ($P_{alv}$) and transalveolar ($P_{ta}$) pressure at functional residual capacity (FRC). Dotted lines show how individual alveoli expand when the lung is inflated to total lung capacity (TLC). (**B**) A spring-mass in-series representation of the lung in panel A. Subpanel (a) and (b) show the system at FRC and TLC, respectively. Springs are extended to different amounts due to the mass elements $M_{i}$ representing regional tissue weight. At the apex (blue), all subtended mass elements act to extend the top spring whereas at the base (orange), only one mass element extends the spring. When the system is stretched to TLC, a common force $F$, representing the change in transpulmonary pressure (${\Delta }P_{tp}$), is applied to the bottom spring.

**Figure 2:**
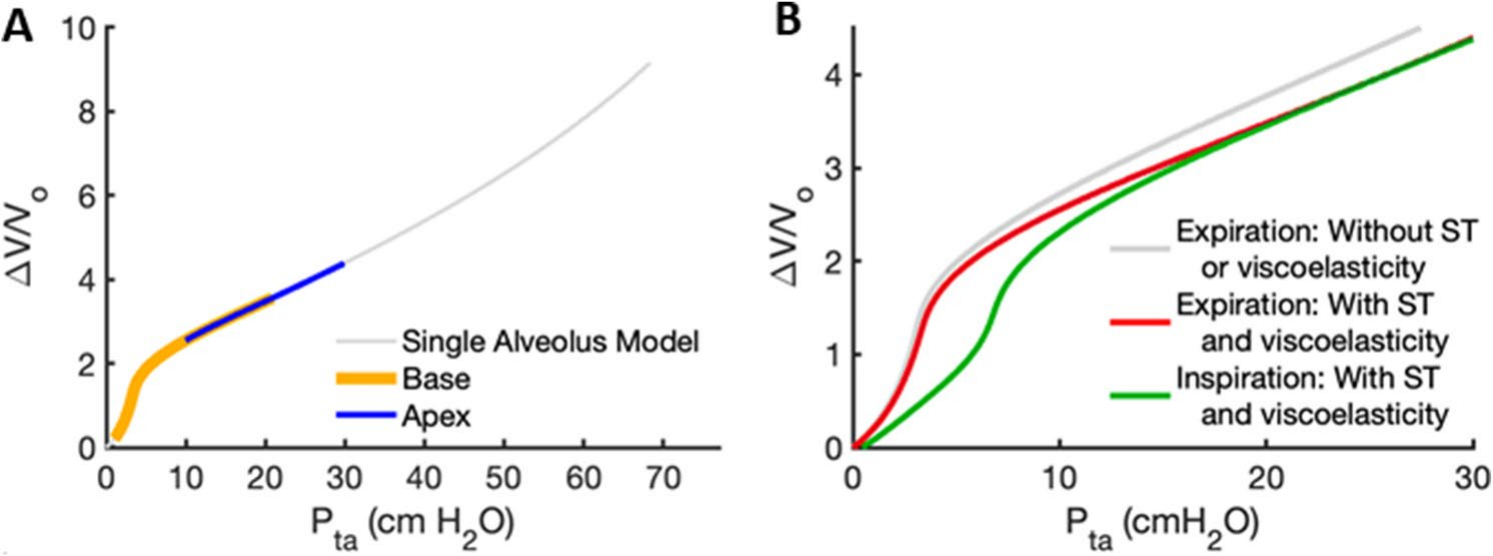
(**A**) The universal pressure–volume (P–V) curve of a single alveolus (gray line) between transalveolar pressures of $P_{ta} =$ 0 and 70 cmH_2_O. The orange and blue segments represent P–V curves of alveoli at the base and apex of the lung, respectively. The alveolus has an inherent minimum volume $V_{0}$ as $P_{ta} \to 0$ cmH_2_O. The volume change ${\Delta }V$, defined such that it is 0 when $P_{ta} = 0$, is normalized by $V_{0}$. (**B**) Single alveolar P–V curves during inspiration (green) and expiration (red) including surface tension (ST) effects as well as viscoelastic collagen and waviness (see main text). For comparison, the deflation P–V curve without surface tension and viscoelasticity is also shown as a gray line.

The equations used for the calculation of alveolar and pleural pressures ($P_{alv}$, and $P_{pl}$, respectively) as well as trans-alveolar pressure ($P_{ta} = P_{alv} - P_{pl}$) are derived in the Methods (Eqs. 11–14). A schematic representation of the distribution of $P_{pl,i}$ through gravitational regions $\left( {i = 1, \ldots ,20} \right),$ and how the model is inflated is depicted in Fig. 1. The full P–V curve of a single alveolus computed using our previously published analytical model^2^ is shown in Fig. 2A. Beyond $P_{ta}$ = $30$ cmH_2_O, the curve displays a slight upward trend, consistent with an increasing compliance (slope of the curve) indicating the onset of inflation instability as demonstrated previously^2^. For $P_{ta} = 0$ cmH_2_O, the volume of a single alveolus was determined to be $V_{0} = 2.4$ nL. This value of $V_{0}$ is then used to normalize the volume change ${\Delta }V$ of the single alveolus P–V curve. The surface tension parameters were determined during deflation ($A = 33, B = 3, C = 0.5$) and inflation ($A = 28.4, B = 0.7, C = 0.305$). Next, a 3-parameter fit to the literature P–V data^22–24^ yielded collagen and elastin effective stiffness values of $Y_{ce} = 1247$ kPa, $Y_{ee} = 18.3$ kPa, respectively, and a lower limit of collagen waviness of $w_{1} = 1.29$ on deflation, whereas these values were $Y_{ce} = 1387$ kPa, $Y_{ee} = 18.3$ kPa, and $w_{1} = 1.2966$ on inflation. Thus, we observe an 11.2% increase in collagen stiffness and a 1.3% increase in the minimum collagen waviness during inflation. These differences in moduli, waviness, and surface tension are reflected in the superimposed expiratory and inspiratory limbs (Fig. 2B). For comparison, the P–V curve without surface tension during expiration is also shown in Fig. 2B. The corresponding functional residual capacity (FRC) on inspiration was calculated to be $2.18$ L.

**Figure 3:**
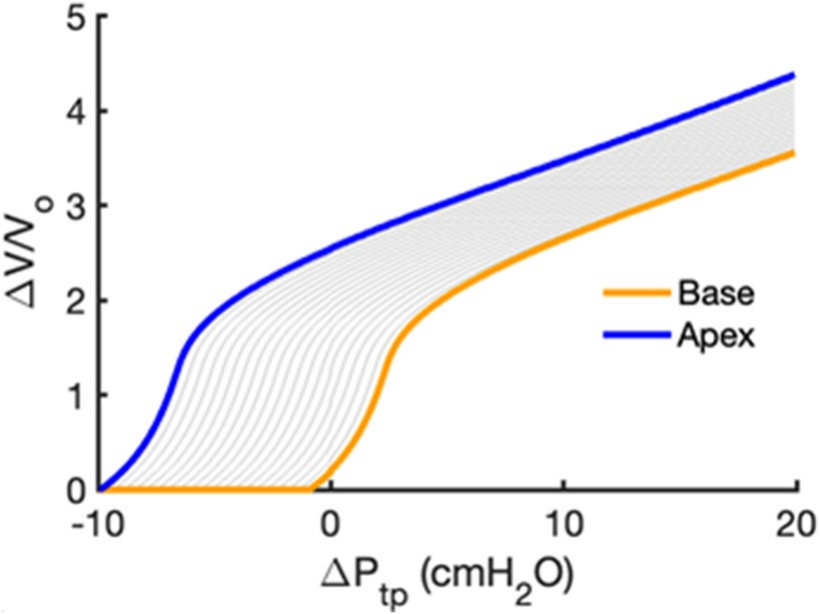
Regional single alveolar P–V curves including the pleural pressure gradient. The P–V curves of the base and apex are shown in orange and blue, respectively, and P–V curves for all other regions by gray lines. The y-axis is the relative volume change as in Fig. 2, whereas the x-axis is given in terms of the common transpulmonary pressure change, ${\Delta }P_{tp}$, which is 0 when the lung is at FRC. Notice that since the pleural pressure at the base at FRC is − 1 cmH_2_O and hence $P_{ta} = 1$ cmH_2_O, a ${\Delta }P_{tp} = - 1$ cmH_2_O creates $P_{ta} = 0$ for which ${\Delta }V$ = 0.

**Figure 4:**
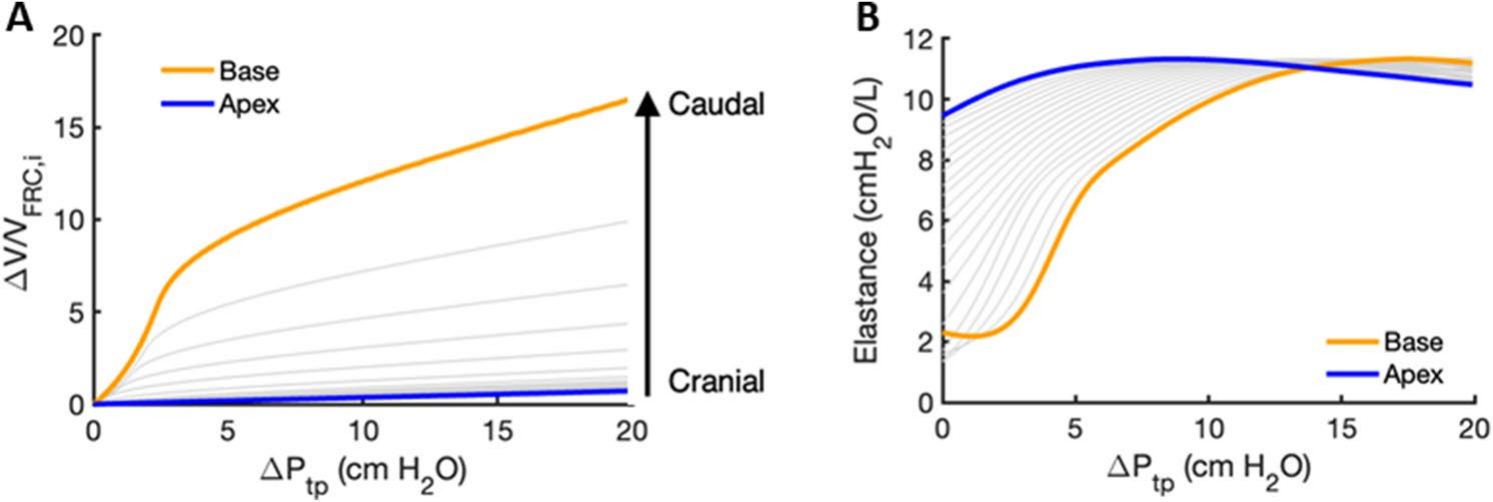
(**A**) Regional P–V curves with the volume change ${\Delta }V$ normalized by the regions own volume at FRC ($V_{FRC,i}$). (**B**) Regional lung elastance as a function of ${\Delta }P_{tp}$. Elastance is defined as the inverse of the slope of the P–V curves in panel (**A**). Base and apex are shown in orange and blue, respectively, while intermediate regions are indicated by the gray lines.

To illustrate how individual alveoli expand in different lung regions, we first note that the single alveolar P–V curve in Fig. 2 is constructed such that ${\Delta }V = 0$ when $P_{ta} = 0$ cmH_2_O. At FRC, the alveoli at the base and apex of the lung were assumed to have $P_{ta,1} = 1$ cmH_2_O and $P_{ta,20} = 10$ cmH_2_O, so they inflated according to the orange and blue segments of the P–V curve in Fig. 2A, respectively. To avoid negative volume changes, we set ${\Delta }V/V_{o} = 0$ for all negative ${\Delta }V/V_{o}$ values, which allowed us to create typical single alveolar P–V curves as a function of vertical distance from the lung base, as shown in Fig. 3. To illustrate, the base of the lung exhibits ${\Delta }V/V_{o} = 0$ when the change in transpulmonary pressure ${\Delta }P_{tp} < - 1$ cmH_2_O corresponding to $P_{ta,1} < 0$. The individual alveolar volumes at FRC depend on their vertical positions in the lung according to the regional distribution of $P_{pl,i} \left( {i = 1, \ldots ,20} \right)$, so the regional P–V curves shown in Fig. 3 are plotted with ${\Delta }V$ normalized by the regional alveolar volume at FRC, $V_{FRC,i} \left( {i = 1, \ldots ,20} \right)$. Also, since $\Delta P_{tp}$ is the common change in transpulmonary pressure that every alveolus in each region experiences during lung inflation, we express the pressure inflating the alveoli in Fig. 3 in terms of ${\Delta }P_{tp}$, where $\Delta P_{tp} = 0$ cmH_2_O at FRC. When the lung is inflated from FRC, alveoli in the apex regions receive significantly less air than those at the base as illustrated in Fig. 4A. The corresponding alveolar elastances, defined as the inverse of the slope of the P–V curve, are shown in Fig. 4B. Alveolar elastance at the apex maintains a high and nearly constant value during inflation, whereas elastance at the base starts low and progressively increases as ${\Delta }P_{tp}$ increases.

**Figure 5:**
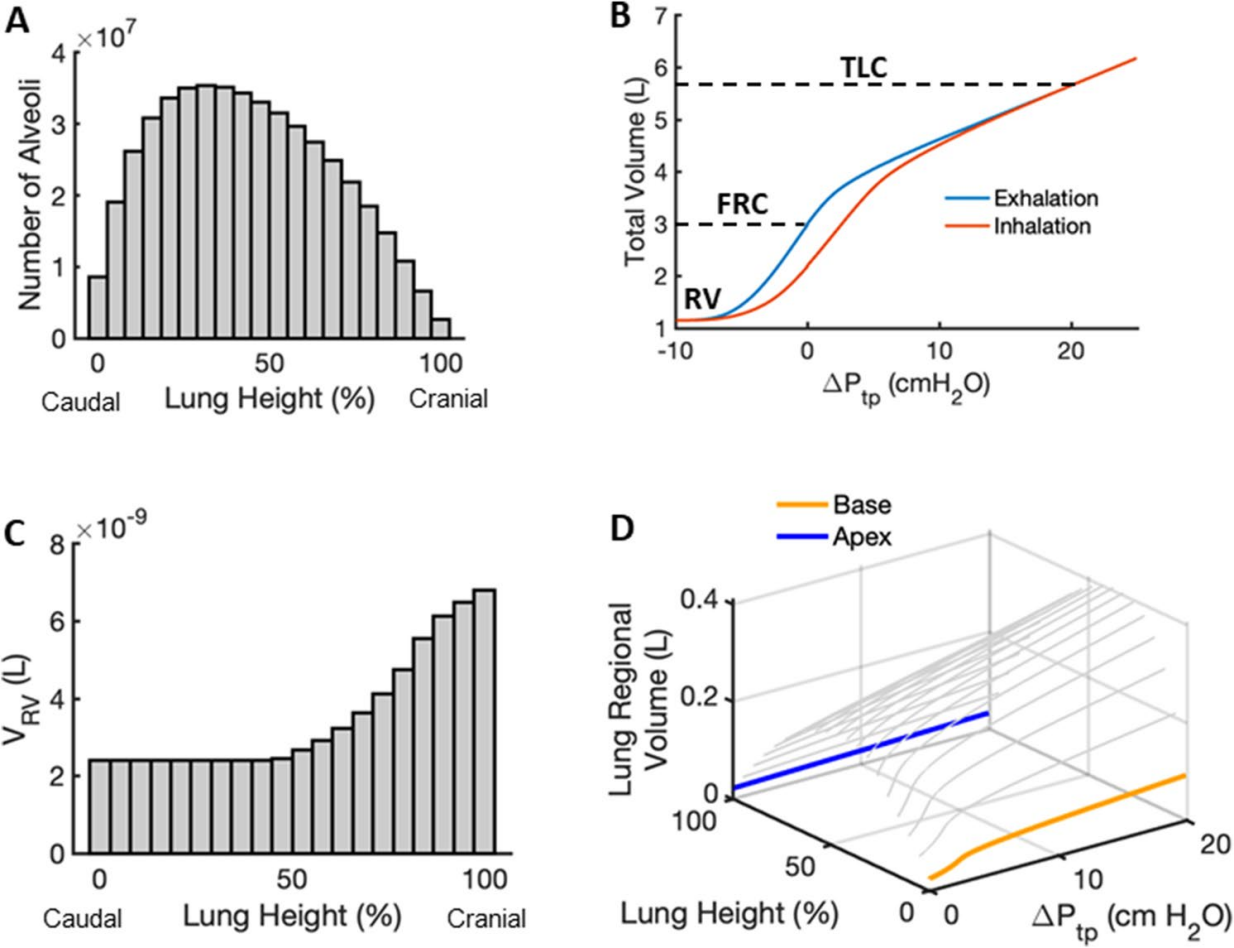
(**A**) Distribution of alveolar numbers in 20 regions versus lung height from base (caudal) to apex (cranial). (**B**) Total lung pressure volume curve during inhalation (red) and exhalation (blue). Residual volume (RV), functional residual capacity (FRC) on the deflation limb, and total lung capacity (TLC), defined respectively as the lung volumes at ${\Delta }P_{tp} = - 7, 0,$ and $20$ cmH_2_O, are also indicated. (**C**) Residual volume distribution versus lung height from base to apex. (**D**) 3D plot of regional lung P–V curves versus lung height from base (orange) to apex (blue).

The apex-to-base distribution of alveolar numbers is shown in Fig. 5A. The total lung P–V curve obtained from this distribution during both inspiration and expiration is shown in Fig. 5B. The residual volume (RV) corresponding to forced expiration from ${\Delta }P_{tp} = 0$ to $\Delta P_{tp} = - 7$ cmH_2_O was determined to be 1.2 L which is close to physiological values^25^. Similarly, total lung capacity (TLC) achieved from inspiration to ${\Delta }P_{tp} = 20$ cmH_2_O is calculated to be $5.64$ L. Note that FRC following inspiration from RV is slightly lower than it is following expiration from TLC due to the existence of P–V hysteresis. As expected, for ${\Delta }P_{tp} = - 7$ cmH_2_O, we find a non-uniform distribution of alveolar residual volumes in which alveoli at the apex remain more open than those at the base (Fig. 5C). Figure 5D shows regional absolute P–V curves from RV to TLC derived from the universal P–V curve of a single alveolus (Fig. 2A), each starting at a different $P_{pl,i}$ due to the gravitational gradient of the intrapleural pressure. These regional P–V curves reflect both the distribution of $P_{pl,i}$ from apex to base as well as the number of alveoli in each region according to Fig. 3A.

**Figure 6:**
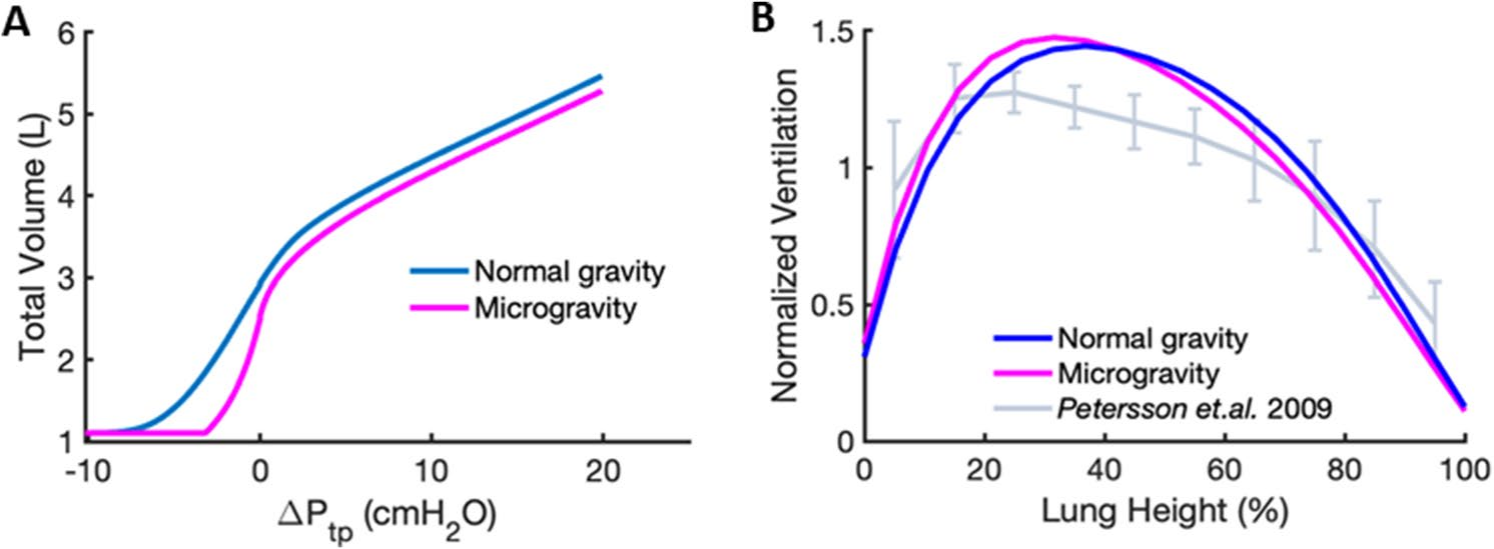
(**A**) Comparison of the deflation limb of the model under normal gravity and microgravity assuming a homogenous distribution of $P_{pl,i} = - 3.25$ cmH_2_O. (**B**) Comparison of model in normal gravity (blue), microgravity (magenta), and experimental (grey) ventilation versus lung height from base to apex at a ${\Delta }P_{tp} = 5$ cmH_2_O.

To further investigate the effects of gravity, we compared the P–V curves between normal 1 g gravity and zero g microgravity environments (Fig. 6A). To mimic the effects of microgravity, we assumed a lung with the same number of alveoli as that in 1 g but having a uniform pleural pressure distribution across all lung regions. We set $P_{pl,i} = - 3.25$ cmH_2_O ($i = 1, \ldots ,20$) at FRC, since this is the pleural pressure on the deflation limb of the P–V curve corresponding to a volume of $2.55$ L. This volume is consistent with the 15% reduction in FRC in microgravity compared to 1 g that has been reported previously^26^. In the microgravity environment, we calculate TLC to be 4.28 L at $\Delta P_{tp} = 20$ cmH_2_O while RV remains the same as in 1 g.

To validate our model, we show in Fig. 6B the model-based regional ventilation evaluated at ${\Delta }P_{tp} = 5$ cmH_2_O as a function of lung height under normal gravity and microgravity simulations. Both curves are compared to measured data obtained by Petersson et al.^27^. All curves are normalized so that the area under them is unity. The shapes of both model-based regional ventilation distributions are similar to those measured experimentally.

**Figure 7:**
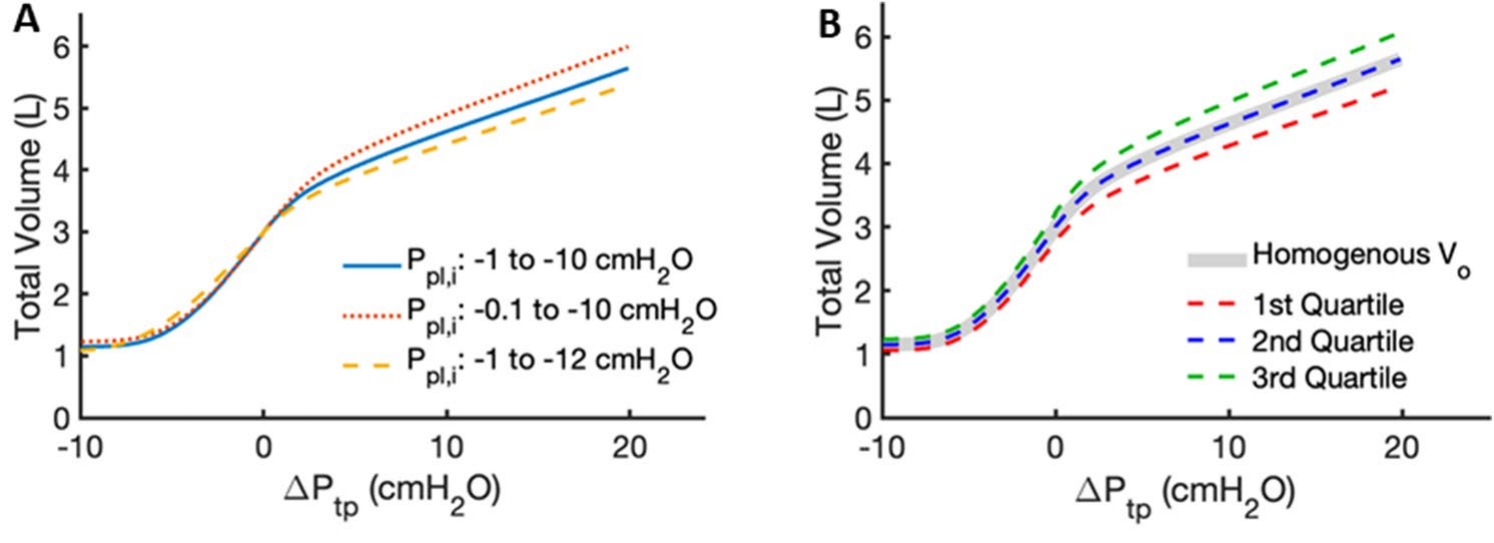
(**A**) Sensitivity of the P–V curve to various pleural pressure distributions. Blue: baseline from − 1 cmH_2_O at the base to − 10 cmH_2_O at the apex; red dotted: from − 0.1 cmH_2_O at the base to − 10 cmH_2_O at the apex; orange dashed: from − 1 cmH_2_O at the base to − 12 cmH_2_O at the apex. (**B**) Sensitivity of the P–V curve to random heterogeneity in alveolar volume $V_{0}$.

The sensitivity of the total lung P–V curve to changes in key model parameters is illustrated in Fig. 7. The effects of changes in the gravity-induced linear gradient in $P_{pl,i}$ are shown in Fig. 7A. By increasing the base pressure $P_{pl,1}$ from $- 1$ to $- 0.1$ cmH_2_O while keeping the apex $P_{pl,20}$ unchanged at − 10 cmH_2_O, we find that TLC increases by $6.0$% from 5.64 to 5.99 L. In contrast, keeping $P_{pl,1}$ unchanged at $- 1$ cmH_2_O while decreasing $P_{pl,20}$ to $- 12$ cmH_2_O causes a $5.4$% decrease in TLC to $5.35$ L. We then investigated how the P–V curve is affected by the reported variability in alveolar size^19^. We assumed that the deflated alveolar volumes follow a uniform distribution between 0.85 $V_{o}$ and $1.15V_{0}$ (Fig. 7B). Repeated simulations show that the median, 1st quartile, and 3rd quartile P–V curves have TLC values of 5.64 L, 5.22 L, and 6.07 L, respectively. The TLC values of the new curves are respectively equal to, $7.4$% below, and $7.6$% above the original TLC which assumed a single value of $V_{o}$.

**Figure 8:**
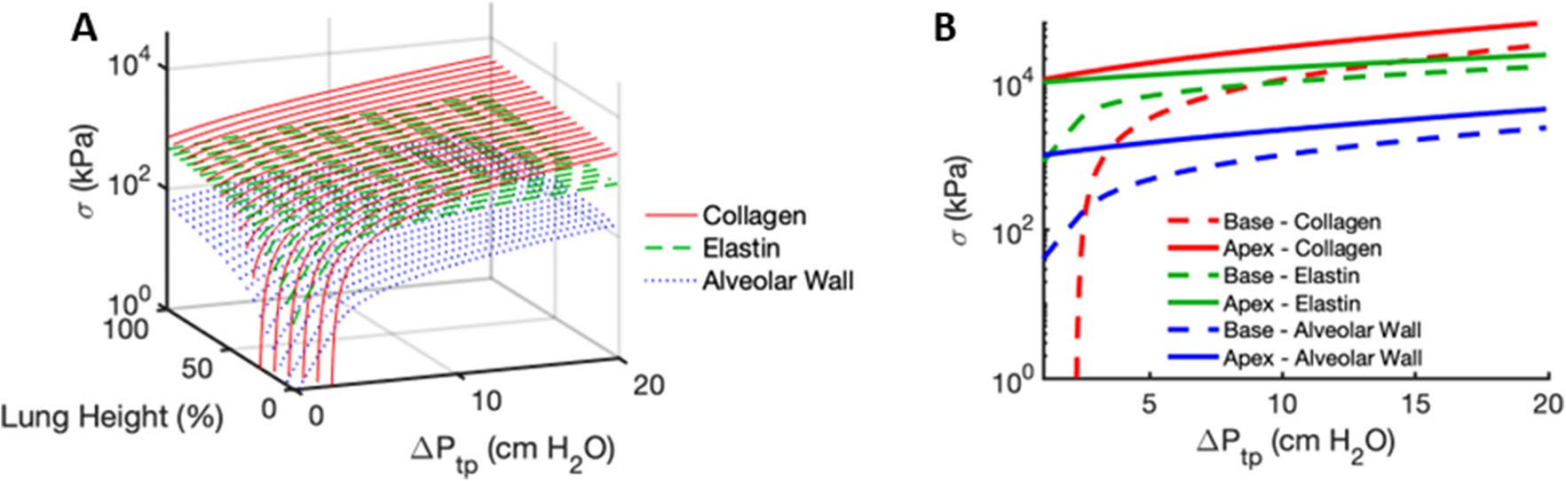
Prediction of the regional circumferential stresses in the alveolar septal walls, on elastin fibers and collagen fibrils. (**A**) 3D plot of the wall stress (blue dotted), elastin stress (green dashed) and collagen stress (red solid) as a function of transpulmonary pressure change above FRC and on height in the lung. (**B**) 2D plot of the same stresses shown only for the base (dashed line) and apex (solid line).

Figure 8 shows the circumferential septal wall and fiber stresses for both collagen and elastin throughout the lung calculated using Eq. (11). At the apex of the lung, the stresses in the alveolar septal walls and their embedded fibers, which are greater than at the lung base at low ${\Delta }P_{tp}$, increase at a much slower rate with $\Delta P_{tp}$ compared to those at the lung base. These differences are due to the gravitational gradient in pleural pressure. The model also predicts that the stress magnitudes span several orders of magnitude; the stresses on the septal wall are smaller than those on the elastin fibers, which are smaller than those on the collagen fibers except at the base at low pressures.

## Discussion

In the normal lung, the gravitational gradient in pleural pressure is a key determinant of regional tissue compliance and alveolar ventilation^25,28^. Previous continuum level models have implemented gravity to predict regional ventilation and ventilation-perfusion matching^29–32^. While such models are useful, they do not account for the critical role of wavy collagen fibers in alveolar mechanics. By applying a height-dependent negative pressure around the outside of our previously described model of a single alveolus^2^, we predict a regional distribution of ventilation (Fig. 6B) that is similar to experimental data^27^ as well as stress distributions in the alveolar walls, and on elastic and collagen fibers (Fig. 8). Furthermore, our model traces these gravitational effects to vertical variations in the behavior of the collagen and elastin fibers that bear most of the mechanical load during breathing. In particular, our results suggest that collagen exhibits a higher elastic modulus and increased waviness during inspiration compared to expiration, as reflected within the superimposed inspiratory and expiratory limbs (Fig. 2B). This result supports findings that conformational changes in extracellular matrix fibers, particularly collagen, contribute to lung tissue viscoelasticity and hence the hysteretic behavior of the lung P–V relationship^21^.

The original single alveolus model consisted of a thick-walled sphere containing linear elastic fibers and linear but wavy collagen fibers, both undergoing large deformations when inflated by a positive alveolar pressure^2^. In the present study, we further assumed collagen to be viscoelastic^33,34^, but elastin to behave as an ideal linear spring^35^ throughout the breathing cycle. With these assumptions, a negative pleural pressure around the outside of the model alveolus (simulating spontaneous breathing) yields the same P–V relationships as applying a positive pressure inside the alveolus (simulating mechanical ventilation). Indeed, despite their differences, Eqs. 1 and 11 provide the same numerical results for the P–V curve of a single alveolus. This is expected also from the symmetry of the mechanical problem since only the pressure differences across the alveolus enter these equations. This also suggests that any difference in lung mechanical or biological behavior stemming from ventilation with negative or positive pressures is likely due to different boundary conditions in the 3-dimensional (3D) shape of the in vivo lung.

Once the symmetry of the alveolar P–V curve was established, we could combine the universal curve (Fig. 2A) from the modified single alveolus model (Eq. 11) with published data on alveolar numbers (Fig. 5A) and a gravitational pleural pressure gradient (Fig. 1A) to obtained regional alveolar P–V curves (Fig. 5D). These curves mimic spontaneous breathing in vivo in the upright human lung due to the gravitational linear gradient that decreases from lung apex to base^36^. However, the pleural pressure gradient in vivo also varies with posture and is different in other species^15^. In addition, due to the complex interactions among the chest shape, abdominal content, muscle contraction and gravity, pleural pressure also varies within each isogravitational plane^16^. Since the model does not incorporate a 3D structure, these effects on pleural pressure, except for those of gravity, were neglected. Accommodating these interactions will allow us to better model lung mechanics in supine, prone, and lateral positions as well as in pathologies that affect the musculoskeletal integrity of the thorax and abdominal pressure.

The linear gradient in $P_{ta}$ in our model also gives rise to vertical variations in $\Delta V/V_{o}$, as shown for the deflation limb of the P–V relationship in Fig. 2A. This causes each lung region to assume a different alveolar volume at FRC, $V_{FRC,i}$, as expected. When deflating to pressures $\Delta P_{tp} < - 1$ cmH_2_O, we assigned all otherwise negative $\Delta V/V_{o}$ values to zero which portrays regional gas-trapping within the alveoli during exhalation (Fig. 3). However, this trapping does not include small airway closure, so alveolar recruitment is not included in the model during inflation. Thus, our model does not reflect the increased compliance seen with alveolar recruitment on inflation, nor can it portray decruitment and consequent atelectasis. The summed minimum volumes of the different lung regions achieved during a maximal expiration yields a value for RV (Fig. 5B) that is similar to reported physiologic values for the normal lung^37^. In addition, as the model lung inflates above FRC, the base receives much larger incremental volumes than the apex (Fig. 4A), achieving a TLC that is also in accord with literature values^38^. The lower volume taken up by the apex compared to the base is largely a consequence of the greater elastance of the apex (Fig. 4B), which in turn is determined by the recruitment of wavy collagen fibers in the alveolar septal walls at lower lung volumes (Fig. 8B). Since the elastance (inverse of compliance) of a lung region scales inversely with the number of alveoli it contains, the distribution of ventilation is somewhat similar to the regional distribution of alveolar numbers (Fig. 6B), in agreement with published data^39^. For example, the region with the lowest elastance is located in the lower-middle of the lung where alveolar numbers are the largest (Fig. 5A). However, not every aspect of regional ventilation can be traced back to gravity; the elastance at the apex starts to decrease slightly for ${\Delta }P_{tp} > 10$ cmH_2_O (Fig. 4B). This is a consequence of geometric inflation instability in which the characteristic stiffening of the P–V curve at high pressure changes due to the alveoli exhibiting a decreasing but positive bulk modulus when inflated above $P_{alv} = 55$ cmH_2_O^2^. Importantly, however, full inflation instability does not occur within the normal physiological range of pressures and lung volumes (i.e., up to $30$ cmH_2_O).

To further elucidate the role of gravity, we compared the P–V curves between normal 1 g and microgravity environments. Under microgravity conditions, we assumed the pleural pressure gradient to be homogeneously distributed. We first used the mean of the pleural pressure range in 1 g as the fixed pleural pressure at FRC in microgravity for all regions. This value of $P_{pl,i} = - 5.5$ cmH_2_O, however, did not result in a lower FRC than in 1 g. Using the pleural pressure corresponding to the height in the lung that bisected the lung mass above and below it into equal portions, $P_{pl,i} = - 4.6$ cmH_2_O, still produced an FRC that was higher in microgravity than at 1 g. The value of pleural pressure that provided a 15% decrease in FRC (2.55 L) consistent with the literature^26^ was $P_{pl,i} = - 3.25$ cmH_2_O. The corresponding RV (1.10 L) and TLC (5.40 L) were respectively 5% and 7% less in microgravity than at 1 g (Fig. 6A). Because the $P_{pl,i}$ in microgravity needed to achieve an FRC consistent with literature values was higher than that of the above two scenarios, this emphasizes the importance of gravity in achieving physiologically relevant lung volumes in normogravity. However, by definition, $V_{0}$ was invariant to microgravity, which is a limitation of the model. Nevertheless, while the absolute magnitudes of lung volumes in the microgravity environment are less than that in 1 g, the shape of the normalized ventilation distribution remains similar between the apex and base (Fig. 6B) confirming the importance of regional alveolar numbers.

We reconstructed the P–V loop of the whole human lung over a complete respiratory cycle (Fig. 5B) by fitting the model to inspiration and expiration separately. The differences between the inspiration and expiration are due to different surface tension-area relationships as well as different Young’s moduli and waviness of the collagen fibers. These factors cause the predicted inspiratory FRC to be lower than the expiratory FRC during cyclic stretching (Fig. 5B). Such an approach to viscoelasticity is consistent with pseudo-elasticity in which the hysteresis loop of the stress–strain curve of a soft tissue is modeled using two different sets of parameters in the strain-energy function to separately account for the loading and unloading limbs of the loop^40^. While this represents rate-independent processes, the experimentally observed differences between inspiration and expiration may also result from extremely long stress-adaptation time constants, which manifest in a near rate-independent viscoelasticity at frequencies surrounding natural breathing. Indeed, such slow stress adaptation has been observed in single collagen fibers^33^. These collagen properties likely contribute to the differences in tissue stiffness between loading versus unloading and are consistent with our model predictions that the inspiratory stiffness of collagen was only $11$% larger than the expiratory stiffness. No data are available concerning the nature of collagen waviness in inspiration versus expiration but our model predicts only a minor difference of $1.3$% in the value of $w_{1}$ that determines the shape of the beta function defining the waviness distribution.

Due to the lack of data, the collagen waviness distribution in the model was assumed to be independent of location in the lung. Since pleural pressure generates significant prestresses in the alveoli at the apex compared to the base, elastin fibers at the apex are more stretched and collagen fibers are partially recruited even at FRC. This in turn results in much higher stresses at the apex at all scales, including septal walls, elastin fibers and collagen fibers (Fig. 8). As inflation proceeds, the differences in stresses at the apex and base are reduced, but at physiological ${\Delta }P_{tp}$ values during normal breathing (i.e., between $0$ and $10$ cmH_2_O) they do not disappear. As a consequence, lung cells are predicted to experience substantially different stresses depending on where the alveolus is positioned along the gravitational axis. For example, epithelial cells attached to type IV collagen of the basement membrane are likely to be exposed to the stresses of the septal wall whereas fibroblast cells that directly adhere to collagen and elastin are more likely to experience stresses that are some combination of the different fiber stresses within the septal walls. Collagen fibers play a critical role in transmitting the transpulmonary pressure to cells, so our model suggests that interstitial cells would be exposed to very different stresses at the apex versus the base of the lung. Previous studies have indeed reported that regional mechanical stress differences in the lung can affect gene expression^41,42^. For example, in a model of unilateral ventilator-induced lung injury in dogs, 1544 genes were differently regulated between the gravitationally dependent and nondependent zones of the lung^43^. Furthermore, when precision-cut lung slices were stretched with patterns mimicking the in vivo dynamics of the apex and the base^44^, the expression of the ACE2 receptor, the entry point for the SARS-CoV-2 virus into alveolar epithelial type II cells^45^, was higher when static stretch was lower suggesting a greater susceptibility to COVID-19-mediated lung injury toward the base of the lung^44^.

Our model is not without limitations. First, extending the single alveolar model to a population of independent alveoli does not account for the fact that neighboring alveoli share the same septal walls, and thus ignores mechanical interactions and load transfer between them. Moreover, we have assumed that heterogeneity in the lung only manifests vertically, and that each horizontal lung section exhibits homogeneous characteristics. These assumptions could be relaxed and the model made more realistic by incorporating horizontal mechanical heterogeneity and by representing the tissue as an alveolar network that transmits stresses between nodes. On the other hand, the sensitivity analysis in Fig. 7 demonstrates that the lung P–V curve is not particularly sensitive to variations in alveolar size or pleural pressure, so the effects of horizontal heterogeneity are likely to be quite small. Our model also does not account for how the shape of the chest wall and the weight of the mediastinal contents, particularly the heart and the blood in the great vessels, may affect pleural pressure gradients. These factors, along with the effects of aging on respiratory muscle function and the dystrophic calcification that stiffens the chest wall, can alter the pleural pressure distribution^46^ and thus impact regional ventilation. Taking such effects into account, however, would require an extension of our currently one-dimensional model into two or three dimensions, which would add a great deal of model complexity. Additionally, no alveolar ducts or airways were implemented in the model, so it is only capable of mimicking quasi-static changes in lung volume. During dynamic breathing, regional variations in airflow resistance can have a major impact on the distribution of ventilation. Indeed, in the normal lung, a significant component of ventilation heterogeneity is due to the fractal nature of the lung structure imposed by the airway tree^47^. The alveoli were also assumed to be perfect spheres, which is certainly an over-simplification that does not allow for prediction of stress variations within a single alveolus. For example, the alveolar entrance ring and the alveolar duct that can be modeled using finite element analysis also modify the stresses^48^. Such stresses have been estimated based on micro-CT imaging and finite element modeling^49^, giving stress values somewhat lower than those in Fig. 8. This discrepancy may be due to the spherical geometry of our model. On the other hand, the assumption of spherical symmetry allows for an analytic solution to our model and thus the computation of the stresses on the fiber systems. Our model does not account for mechanical interactions between the elastin and collagen fiber system^50^. Despite these limitations, however, our model provides novel insight into how the total lung P–V curve arises from gravity acting on the distributed mechanical properties of individual alveoli as determined by fiber stresses within the septal walls in the human lung. Finally, collagen waviness was assumed to be homogeneous in the lung. Since the mechanical stresses experienced by cells can have an important impact on mechanotransduction, one might speculate that alveolar septal wall thickness and collagen waviness are adjusted during development to improve the homogeneity of the stresses experienced by cells embedded in the lung parenchyma. This warrants further experimental study.

In conclusion, we have incorporated a gravity-induced pleural pressure gradient into a previously established model of a single alveolus during inflation to predict how alveolar P–V relations vary spatially throughout a normal upright human lung. By empirically accounting for dynamic surface tension and tissue viscoelastic behavior, we have been able to generate a P–V hysteresis loop reminiscent of those observed during breathing. The model links the P–V loop to the stresses on the collagen and elastin fibers that comprise the load-bearing elements of the alveolar septal wall, and how these stresses are distributed throughout the lung. These predictions can help us better understand how the mechanical stresses arising from breathing may affect the cells embedded within the lung tissue, with implications for mechanotransduction in both healthy and diseased states.

## Methods

### Analytical model of an individual alveolus

The details of our model of the P–V relationship of a single spherical thick-walled alveolus are provided in our previous publication^2^. Herein, the term “P–V” is used to describe the general concept of pressure–volume relations but is nonspecific for the pressure terms that we define properly later. Briefly, we consider the inflation of a sphere having walls of finite thickness composed of a mesh of circumferential non-interacting elastic fibers and collagen fibers. The elastin fibers are under tension and thus form smooth curves aligned with the great circles on the sphere. The collagen fibers are similarly arranged but at functional residual capacity (FRC) they are not under tension and thus form wavy paths along the great circles. The degree of waviness of a fiber along its circumferential path determines how much the model alveolus must be inflated above FRC before the fiber becomes straight and begins to bear stress. The distribution of collagen fiber waviness determines how these fibers are recruited as the alveolus is inflated, and thus has a defining influence on the nature of the alveolar P–V relationship.

The alveolar tissue has point symmetry through the center of the sphere and thus expands uniformly. At the baseline alveolar volume, $V_{0}$, the distance from the alveolar center to a point within the wall is the radius $R$ such that $R_{i} \le R \le R_{e}$, where $R_{i}$ and $R_{e}$ are the radii at the inner and outer surfaces, respectively, defining the wall thickness as $R_{e} - R_{i}$. The corresponding quantities in the inflated state are $r_{i} \le r \le r_{e}$. The circumferential stretch ratio of the tissue at radius $r$ is $\lambda = \frac{r}{R};$ thus, $\lambda_{i} = \frac{{r_{i} }}{{R_{i} }} \le \frac{r}{R} \le \frac{{r_{e} }}{{R_{e} }}$. A thickness mapping parameter is defined in the undeformed state as $S = \frac{R}{{{ }R_{i} }}$ and spans the alveolar wall from its inner surface where ${ }S_{i} = \frac{{{\text{R}}_{i} }}{{{ }R_{i} }} = 1$ to the outer surface where ${ }S_{e} = 1.05$. The alveolar sphere is inflated by an internal alveolar pressure, $P_{alv}$, that is positive relative to the zero outside pressure according to^2^

1 $$P_{alv} = \left( {\sigma_{c} + \sigma_{e} } \right)\left[ {\left( {\frac{{\lambda_{i}^{3} + S_{e}^{3} - 1}}{{\lambda_{i}^{3} }}} \right)^{2/3} - 1} \right] + P_{s}$$

where $\sigma_{c}$, and $\sigma_{e}$ are the mechanical stresses carried by collagen fibers, and elastin fibers, respectively, and $P_{s}$ is the pressure due to alveolar surface tension, $\gamma$ defined below. The expressions for the circumferential stresses $\sigma_{c}$ and $\sigma_{e}$ depend on the elastic moduli of collagen and elastin, respectively, and the waviness ($w$) of the collagen. These expressions were derived previously^2^. Briefly, the circumferential stress in the alveolar wall, at any given layer, due to elastin fibers is:

2 $$\sigma_{e} \left( {\lambda ,S} \right) = Y_{ee} \frac{\lambda }{S}\left( {\lambda - 1} \right)$$

whereas that due to the wavy collagen fibers is:

3 $$\begin{aligned} \sigma_{c} \left( {\lambda ,S} \right) & = \frac{{Y_{ce} }}{{B\left( {\alpha ,\beta } \right)\alpha \left( {\alpha + 1} \right)}}\frac{\lambda }{S}\frac{{\left( {\lambda - w_{1} } \right)}}{{w_{1} }}\left( {\frac{{\lambda - w_{1} }}{{w_{2} - w_{1} }}} \right)^{\alpha } \left( {\left( {\alpha + 1} \right)A_{1} - \alpha B_{1} } \right), \\ & \quad w_{1} \le \lambda < w_{2} \\ \end{aligned}$$

4 $$\sigma_{c} \left( {\lambda ,S} \right) = \frac{{Y_{ce} }}{{B\left( {\alpha ,\beta } \right)\alpha \left( {\alpha + 1} \right)}}\frac{\lambda }{S}\frac{{\left( {\lambda - w_{1} } \right)}}{{w_{1} }}\left( {\left( {\alpha + 1} \right)A_{2} - \alpha B_{2} } \right), \lambda \ge w_{2}$$

where *A*_*1*_*, B*_*1*_*, A*_*2*_*,* and *B*_*2*_ are calculated from the Appell hypergeometric function of two variables (*α* and *β*). Additionally, $Y_{ce}$ and $Y_{ee}$ are the effective fiber elastic moduli defined as *Y*_*ce*_ = *Y*_*c*_* δ*_*A,c*_ and *Y*_*ee*_ = *Y*_*e*_* δ*_*A,e*_ for collagen and elastin, respectively, with *δ*_*A,c*_ and *δ*_*A,e*_ being the total area fractions and $Y_{c}$ and $Y_{e}$ the absolute moduli for collagen and elastin, respectively. The parameters $w_{1}$ and $w_{2}$ are the lower and upper limits, respectively, of the waviness distribution, which is a beta distribution with parameters *α* and *β*.

Next, we derive an equation for the case of negative pressure-driven inflation. Recalling Eq. 5.56 from our previous work^2^, the radial stress is given as a function of $\lambda_{i}$ and $S$ as follows:

5 $$\sigma_{rr} \left( {\lambda_{i} ,S} \right) = \frac{2}{{\left( {S^{3} + \lambda_{i}^{3} - 1} \right)^{2/3} }}\mathop \int \limits_{1}^{{{\text{S}}_{e} }} \frac{{S^{2} }}{{\left( {S^{3} + \lambda_{i}^{3} - 1} \right)^{\frac{1}{3}} }}\sigma_{\theta \theta } \left( {\lambda_{i} ,S} \right)dS$$

where the total circumferential Cauchy wall stress $\sigma_{\theta \theta } = \sigma_{c} + \sigma_{e}$. To formulate the negative pressure inflation of the alveolus, we define the boundary conditions to be $P_{alv} = 0$ and the pleural pressure $P_{pl}$ as the negative of the radial stress at the outer surface of the sphere. Since the transalveolar pressure is defined as

6 $$P_{ta} = P_{alv} - P_{pl} = - P_{pl} > 0,$$

the transalveolar pressure due to fibers alone is given by:

7 $$P_{ta,f} = \sigma_{rr} \left( {\lambda_{i} ,S_{e} } \right) = \frac{2}{{\left( {S_{e}^{3} + \lambda_{i}^{3} - 1} \right)^{2/3} }}\mathop \int \limits_{1}^{{{\text{S}}_{e} }} \frac{{S^{2} }}{{\left( {S^{3} + \lambda_{i}^{3} - 1} \right)^{\frac{1}{3}} }}\sigma_{\theta \theta } \left( {\lambda_{i} ,S} \right)dS$$

By assuming a thin but finite thickness, we can approximate $P_{ta,f}$ as:

8 $$P_{ta,f} = \frac{2}{{\left( {S_{e}^{3} + \lambda_{i}^{3} - 1} \right)^{2/3} }}\overline{\sigma }_{\theta \theta } \mathop \int \limits_{1}^{{{\text{S}}_{e} }} \frac{{S^{2} }}{{\left( {S^{3} + \lambda_{i}^{3} - 1} \right)^{\frac{1}{3}} }}dS$$

where $\overline{\sigma }_{\theta \theta }$ is the analytic approximation of the fiber-related wall stress at the middle layer of the wall^2^. The integral can be calculated by introducing a new variable

$$u = \left( {S^{3} + \lambda_{i}^{3} - 1} \right)^{1/3}$$

which gives the following result:

9 $$P_{ta,f} = \frac{2}{{\left( {S_{e}^{3} + \lambda_{i}^{3} - 1} \right)^{\frac{2}{3}} }}\overline{\sigma }_{\theta \theta } \frac{1}{2} \left[ {\left( {S^{3} + \lambda_{i}^{3} - 1} \right)^{\frac{2}{3}} } \right]_{1}^{{S_{e} }}$$

Substituting the limits and rearranging, we obtain the final form:

10 $$P_{ta,f} = \overline{\sigma }_{\theta \theta } \left[ {1 - \frac{{\lambda_{i}^{2} }}{{\left( {S_{e}^{3} + \lambda_{i}^{3} - 1} \right)^{2/3} }}} \right]$$

Finally, the total transalveolar pressure is the sum of the fiber and surface tension pressures:

11 $$P_{ta} = \left( {\sigma_{e} + \sigma_{c} } \right)\left[ {1 - \frac{{\lambda_{i}^{2} }}{{\left( {S_{e}^{3} + \lambda_{i}^{3} - 1} \right)^{2/3} }}} \right] + P_{s}$$

Finally, the surface tension term in Eqs. 1 and 11 is defined as^51^

12 $$P_{s} = 2\frac{{{\upgamma }\left( {r_{i} } \right)}}{{r_{i} }} = \frac{{2\gamma \left( {{\Delta }V/V_{o} } \right)}}{{R_{i} \left( {\frac{\Delta V}{{V_{o} }} + 1} \right)^{1/3} }}$$

where ${\Delta }V = V_{alv} - V_{o}$. Thus, $\gamma$ depends on ${\Delta }V$ according to the following 3-parameter sigmoidal relationship

13 $$\gamma \left( {{\Delta }V/V_{o} } \right) = \frac{A}{{1 + {\text{exp}}\left( { - \frac{{\frac{\Delta V}{{V_{o} }} - B}}{C}} \right)}}$$

where the parameters $A, B$ and $C$ were independently determined for both inspiration and expiration by fitting Eq. (12) to experimental data reported in a previous study^52^. To construct the alveolar P–V curve, alveolar volume, $V_{alv}$ is defined as

14 $$V_{alv} = V_{0} \lambda_{i}^{3}$$

which increases from its initial value of $V_{0}$, and is defined accurately below. Thus, Eqs. 11–14 can be used to obtain alveolar P–V curves starting from different values of $P_{pl}$ according to the gravitationally-induced pleural pressure gradient in the in situ lung.

### Model parameters

Baseline parameters were obtained by fitting the model to deflation P–V curves. Using second harmonic generation imaging, $w$, defined as the contour length over the arch length, of the collagen fibers was found to follow a 4-parameter ($\alpha ,\beta ,w_{1} ,w_{2}$) beta distribution ($\alpha = 1.89$, $\beta = 3.60$) with minimum and maximum waviness values of $w_{1} = 1.12$ and $w_{2} = 2.53$, respectively^2^. By performing a 3-parameter fit of the model defined by Eqs. 11–14 to the deflation limbs of previously obtained human P–V curves^22–24^. Using the parameters from these fits, $P_{alv}$ can be plotted against $V_{alv}$ to predict the P–V curve of a single alveolus. To extend the single alveolus model of Eq. 11 to cyclic inflation and deflation mimicking breathing, we fit the surface tension expression (Eq. 12) to both expiratory and inspiratory data collected previously^52^. Similarly, to account for collagen viscoelasticity, we performed a 2-parameter fit of our model, including $Y_{ce}$ and $w_{1}$, to the inflation limbs of human P–V data^10,22^. Since tropoelastin as well as the elastic fibers are nearly perfectly elastic^35^, we assumed elastin to behave as an ideal linear spring throughout the breathing cycle. We used the expiratory limb of the P–V curve to perform our calculations unless otherwise specified.

### Modeling the effects of gravity on the lung

We divide an upright-oriented lung from base to apex into $n = 20$ regions of equal height. The average alveolus within the $i$th region exhibits a unique P_alv_-V relationship due to differences in the local value of the pleural pressure ($P_{pl,i}$) in that region $i$. Since the local trans-alveolar pressure in region $i$ is $P_{ta,i} = - P_{pl,i}$, regional P_alv_-V curves initiated from the functional residual capacity (FRC) for each of the $n$ lung regions can be generated from the universal alveolar P_ta_-V curve given by Eq. 11 by properly defining the values $P_{pl,i} \left( {i = 1, \ldots ,n} \right)$.

Based on reports of the effects of gravity on $P_{pl}$ ^15^, we assumed that at FRC the alveoli at the base of the lung in region $i = 1$ are subject to a $P_{pl,1}$ of $- 1$ cmH_2_O, while alveoli at the apex in region $i = 20$ experience a $P_{pl,20}$ of $- 10$ cmH_2_O, with a linearly increasing gradient of pleural pressure for the intermediate regions^36^. We note, however, that the $P_{pl}$ gradient can vary based on body positioning^15,53^.

A schematic representation of the distribution of $P_{pl,i}$ and lung inflation is depicted in Fig. 1A for an illustrative example of our model comprised of four vertically stacked regions. We define $V_{FRC,i}$ as the average volume of an alveolus in region $i$ distended by $P_{ta,i} = - P_{pl,i}$ when the lung is at FRC. Similarly, we define $V_{RV,i}$ and $V_{TLC,i}$ as the average volumes of alveoli in region $i$ when the lung is at residual volume (RV) and total lung capacity (TLC), respectively. Furthermore, we define $V_{o}$ as the maximally deflated volume of an alveolus of an excised lung, which is also the volume reached when $P_{ta,i} = 0$ cmH_2_O. Note that depending on the vertical location of an alveolus, its $V_{TLC,i}$, $V_{FRC,i}$, and $V_{RV,i}$ are different to those of other alveoli above or below it. However, $V_{o}$ is an intrinsic property of alveolar tissue mechanics and is thus independent of $i$.

Conceptually, our model can also be portrayed as a series of nonlinear springs and mass elements suspended vertically (Fig. 1B). At FRC (Fig. 1B, panel a), the length $x_{FRC,i}$ of spring $i$ represents the volume $V_{FRC,i}$ of alveolus $i$. $x_{FRC,i}$ is determined by the force $F_{FRC,i} = \mathop \sum \limits_{j = 1}^{i} M_{j} g$ ($M_{j}$: mass of region $j$; $g$: gravitational acceleration) due to the sum of the weights of all mass elements beneath spring $i$, together with the local pleural pressure $P_{pl,i}$, via Eq. 11. As an additional force $F$, representing a change in transpulmonary pressure (${\Delta }P_{tp}$), is applied at the end of the chain, the spring system stretches in the direction of $F$ (Fig. 1B, panel b shows the stretch corresponding to TLC). Each spring elongates by a different amount $\Delta x_{i}$ based on its nonlinear spring constant $k_{i}$, which in turn depends on $x_{FRC,i}$, again via Eq. 11. The sum of the changes in the lengths of all the springs corresponds to the change in total lung volume from FRC to TLC. This system thus mimics how a global $\Delta P_{tp}$ inflates each alveolus to different volumes ${\Delta }V_{i}$ according to their individual elastances, defined as the inverse of the slope of the P–V curve.

The sum of the changes in spring lengths due to the application of $F$ corresponds to the change in total lung volume from FRC. For example, for a $\Delta P_{tp}$ of $20$ cmH_2_O, the change in total lung volume leads to TLC as shown in panel (b). Thus, given the $P_{pl,i}$ distribution for all $i = 1, \ldots ,n$, we first calculated the individual alveolar $V_{FRC,i}$ values from Eq. 11. Next, the individual volume changes ${\Delta }V_{i}$ were computed also from Eq. 11 due to ${\Delta }P_{tp}$. In other words, just as the spring system was stretched by a single force $F$, our distributed alveolar model was inflated by a common ${\Delta }P_{tp}$ superimposed on the individual $P_{pl,i}$ distribution. For the spring system, the final length is obtained by summing the displacements for all springs.

### Calculating regional and total lung P–V curves

Both the regional and total volumes in the model were scaled to correspond to physiological values. To do this, we first calculated how the number of alveoli varies as a function of vertical position in the lung assuming a constant tissue mass for all alveoli^54^. On expiration, based on a total volume for the lung at FRC of 3 L for a healthy human adult, this provided the numbers of alveoli in each of the 20 vertical lung regions as well as in the lung as a whole. These numbers were then used to calculate both expiratory and inspiratory P–V curves over the vital capacity range using the above equations. We also back-calculated $V_{o}$ to estimate $V_{RV,i}$ for each lung region at a $\Delta P_{tp}$ of $- 7$ cmH_2_O. Note that $V_{RV,i}$ is the alveolar volume in a lung after forced expiration in vivo and hence can vary regionally. Taking the summation of all $V_{RV,i}$ allows us to calculate the RV of our model. Similarly, we can sum the $V_{TLC,i}$ to find the TLC. These P–V relationships assumed quasi-static inflation and deflation of the lung in which there is complete equilibration between the alveolar and atmospheric pressure at all times^15^.

### Regional stresses on collagen and elastin

Analyzing the separate contributions of $\sigma_{c}$, and $\sigma_{e}$ to $P_{ta}$ in Eq. 11, we calculated the circumferential stresses $\sigma _{{\theta \theta ,c}}$ and $\sigma _{{\theta \theta ,e}}$ in the collagen and elastin fibers, respectively. Dividing these stresses by the respective collagen and elastin volume fractions of $0.08$ and $0.05$^55^ provided the absolute stresses per fiber as a function of vertical position in the upright lung. Note that $0.08$ and $0.05$ are the volume fractions, but assuming fibers running parallel to the alveolar wall, they can be estimated to be equal to area fractions.

## Data Availability

All data generated or analyzed in this study are included in this article.
